# Supplementary material for: A Qualitative Study of Parents’ Conceptualizations on Fever in Children Aged 0 to 12 Years
Source: Int J Environ Res Public Health. 2019 Aug 16;16(16):2959. doi: 10.3390/ijerph16162959 (PMC6720744; doi:10.3390/ijerph16162959)
Supplement: Supplementary file 1 [file ijerph-16-02959-s001.pdf]

Table S1. Relationship between themes, categories, subcategories and codes.

THEME	CATEGORY	SUBCATEGORY	CODE	VERBALIZATION
Knowledge/ beliefs of parents	Incidence		"being prone or not"	"He is not prone to getting a fever" (Mother I; GNHPM, UE)
regarding fever	Causes of fever		Chance	"My child, very seldom, she has been lucky" (Father f; GNHPF, RE)
			Going to nursery school	"They haven't given me any problems except when they have gone to nursery school, they have been a bit more continuous, one after the other." (Mother ß; GHPM, RE)
			Growth	"He is growing, so he has a fever" (Father b; GNHPF, RE)
			Weaning	"I was breastfeeding until the age of 2, and she almost didn't have fever the first years" (Father o, GHPF, RE)
			Tonsillitis/ throat	"She had fever constantly because she had enlarged tonsils… the tonsils were operated on and all the problems went away" (Mother E; GNHPM, RE)
			Ear	"He gets a fever, normally it's because of the ear" (Mother C; GNHPM, RE)
			Cold/ runny nose	"At the beginning you always think it's a cold" … "maybe you see your child with a runny nose" (Father i; GHPF, UE)
			Teething	"When they begin teething" (Father f; GNHPF, RE)
			Unknown source	"It didn't reveal itself" (Mother B; GNHPM, RE)
			Reaction to	"We have been told that vaccines give them fever" (Father u; GNHPF,

	vaccines	UE)
	Infection by virus/ bacteria: chicken pox, meningitis, scarlet fever, hand, foot and mouth disease, mononucleosis	"It was a virus with fever" … "then he started with rashes and he had hand foot disease" (Mother W; GHPM, RE)<br>"It must be a virus" … "chickenpox" … "we associated it with meningitis" (Father b; GNHPF, RE)<br>"The first thing I think, to attempt to associate it, something viral without any importance" (Father n; GHPF, UE)
	Urinary infection	"When they were younger maybe a urinary infection" (Mother V; GHPM, RE)
	Excessive heat	"A heatstroke" (Father u; GNHPF, UE)
	Tiredness	"At times I believe that even tiredness leads to fever" (Mother D; GNHPM, RE)
	"Getting worked up"	"From being startled, the fever appeared" … "having some fever, but because of getting worked up" (Mother F; GNHPM, RE)
Classification of fever	No fever	"37 [degrees] was not considered fever." (Mother B; GNHPM, RE)
	Low fever/ "normal fever"	"It really depends on what each of us has in our head regarding what normal fever is, you know, 37 [degrees] and a bit, 38 [degrees]" (Father x; GNHPF, UE)
	High fever	"Besides, high fever, of 39 [degrees]" (Mother Q; GHPM, UE)
	Very high fever/ "Fever spike"	"She got very ill, with a very high fever, 40 [degrees] fever…. It was like a super high fever" (Mother A; GNHPM, RE)

	Prevention	Immunization	Contagion	“One of them becomes one child is much more ill than the others and then they all fall ill….” (Mother M; GNHPM, UE)
			Vaccines	“There are many illnesses which were eradicated from this country and now foreigners have come who have not been vaccinated and all, and once again illnesses are coming back, many of them with fevers (Father d; GNHPF, RE)
		Prophylaxis	Covering the throat/ears	“You take him around with a neck warmer to protect his neck, for him, just in case, his throat is weaker and that’s that.” … “Perhaps I act more based on prevention, we have to be careful of that, if they are weak in the ears, then they need ear muffs...” (Mother C; GNHPM, RE)
			Probiotics	“For prevention purposes, later they sent me some little sachets which were probiotics” (Mother D; GNHPM, RE)
			Appropriate diet	“I believe that eating well and staying strong… isn’t associated with having a high fever.” (Father d; GNHPF, RE)
	Tolerance to fever		Good	“Really, the boy wasn’t bad, he didn’t look bad… but his fever was 39 [degrees]” (Mother B; GNHPM, RE)
			Poor	“She has a hard time when she has fever” (Father v; GNHPF, UE)
	Symptoms		Singularity of symptoms	“Each child is in their own world” (Mother C; GNHPM, RE)
			General malaise	“They start with fever, with that discomfort (Father i; GHPF, UE)
			Headache	“Her head ached a bit” (Mother Q; GHPM, UE)

Arthralgia	“He had aches in all parts of the joints, joint pain in the bones, they don’t like you to touch them, they are more sensitive” (Father u; GNHPF, UE)
Crying	“If the child has a fever they will wake up crying” (Mother J; GNHPM, UE)
Hot body	“The heat they give off” (Mother γ; GHPM, RE)
Distal temperature: hot/cold	“I see the fever because her hands get hot and the feet are burning due to the fever” (Mother F; GNHPM, RE)<br>“The hands start to get cold, that is when the fever is going to start going up” (Mother J; GNHPM, UE)
Red cheeks	“They get red cheeks” (Mother ß; GHPM, RE)
Red ears	“I notice because the ears get very red, very red… When this area is red then I know there is a fever.” (Mother P; GHPM, UE)
Glassy eyes	“She gets little tears” (Father e; GNHPF, RE)
Bags under eyes	“When he gets fever, he gets bags under his eyes” (Mother α; GHPM, RE)
Goosebumps	“His hair stands on end” (Mother J; GNHPM, UE)
Shivering	“Normally when he has a high fever, he gets, well, we all get shivers” (Father z; GNHPF, UE)
Lack of appetite	“Because he stopped eating, his fever went up” (Mother D; GNHPM, RE)
Vomiting	“My children vomit a lot with fever, then it becomes hard to get their

			fever down" (Mother T; GHPM, UE)
		Irritability/ unrest	"I notice her fever when she is very agitated, when she is very irritated" (Mother F; GNHPM, RE)
		Sleepiness/ malaise	"I see her a bit down in spirits, she is very active and you can tell immediately, she is very active and she becomes more dull", "you can tell she is less active" (Father w; GNHPF, UE)
		Acting up	"He gets a bit silly" (Mother J; GNHPM, UE)
		Seeking nurture from the mother	"My wife finds it tedious, the little one has her fed up… He is like, 'mama, can you pick me up? And [calling] mama, mama… it is complete momism" (Father u; GNHPF, UE)
Evolution		Duration: 2-3 days	"It is three days of fever going up and three days of fever going down" (Mother D; GNHPM, RE and Mother L; GNHPM, UE)
		Fever variations during the day	"Primarily, we all know that at night fever goes up more" (Father x; GNHPF, UE)
		Spontaneous resolution/ self-limited process	"Today he gets up with a fever and tomorrow he may not have it… these are viral processes which, just as they come, they go" (Mother Z; GHPM, RE)
Repercussion of fever on health	Beneficial	Defense	"A normal reaction that he is fighting a virus and he gets the fever that he is fighting off" (Mother J; GNHPM, UE)
		Warning sign	"A fever is something that tells us there is something bad" (Father g; GNHPF, RE)
		Development	"Something physiological, quite normal in development" (Father r;

				GHPF, RE)
		Harmful complications of fever	Febrile convulsions	“Fever of more than 40 [degrees] ... because he may start to convulse” (Mother M; GNHPM, UE)
			Brain damage/ mental problems	“At the level of the brain it can leave some type of aftereffect” (Mother γ; GHPM, RE)
			Protein/cell denaturation	“After 40 [degrees] there is protein denaturation, that is the dangerous thing...” (Mother L; GNHPM, UE)
			Death	“You also wonder about it [death], the repercussions, you know?” (Father k; GHPF, UE)
Parents sentiments regarding their child’s fever	Positive	Satisfaction	Temperature decrease	“I feel satisfied with it going down, maybe if the fever is at 38.8 [degrees] and it goes down to 38 [degrees], well really he appears much better” (Mother Y; GHPM, RE)
		Reassurance	Resolution of fever	“Sincerely, I breathe again when it resolves” … “I felt so reassured, when it is under control” (Mother C; GNHPM, RE)
			Health center close by	“Having it so close [the hospital], more at hand, gives you peace of mind” (Father t; GNHPF, UE)
			Help of grandparents	“The grandparents calm you down a lot I think, if you get there with the boy, and you are more fearful, they tell you not to worry, water and that’s it. They take it differently” (Father x; GNHPF, UE)
	Negative	Concern/anxiety/ fear	Unknowing the cause	“I feel overwhelmed not knowing what is causing the fever” … “My concern was that, what is causing the fever” … “A terrible fright because I didn’t see the problem where the fever was coming from” (Mother K; GNHPM, UE)

At very high temperatures	"Fear when they have high fever" … "If he gets 39, 40, 41 [degrees] well …that is my fear" (Father a; GNHPF, RE)
Excessive duration	"Feeling distressed... if the fever lasts more than 3 days" (Mother Y; GHPM, RE)
Administration of antipyretics	"Often I have also felt fear at times when I have given him some kind of medication and his temperature has gone down abruptly lower than normal" (Mother γ; GHPM, RE)
Resistance to treatment	"The problem is not that it goes down, it's that you give him something and it doesn't go down, or it goes down only a bit, things like that, you keep seeing him with the discomfort, in my case, at least in that situation… I get worried" (Father i; GHPF, UE)
Fever complications	"That is the fear, that she will start to convulse" (Father v; GNHPF, UE)
Severe illnesses	"Horrified of meningitis, pneumonia" (Mother A; GNHPM, RE)
Inversely proportional to the number of children	"With the first child I think that you get a bit more overwhelmed… I think that anything gets to you, but now, with my second girl I was less worried" (Mother ß; GHPM, RE)
Inversely proportional to the age	"When they are smaller you get more scared" (Mother L; GNHPM, UE)
Night-time	issues

“You are scared of how your night is going to be” … “You fear that perhaps the fever will get too high and that you are sleeping and you don’t notice, during the day you are more watchful” (Father u;

		GNHPF, UE)
	Responsibility in care	"In health matters, it's all up to me, which is what gives me anxiety … it really makes me feel overwhelmed, he is like, 'you are the nurse, here you go, your son has a fever or whatever, do something" (Mother O; GHPM)
	Only one carer	"At the beginning, for example, when the girls were born, I went away for 10 months because I lost my job. My parents-in-law came, I think it was for 5 months because we were terrified about that [leaving my wife alone]" (Father e; GNHPF, RE)
	"Dire anguish"	"You feel dire anguish" (Mother N; GNHPM, UE)
	Lack of negative emotions	"We aren't the type to worry a lot, or feel scandalized by fever" "We are not scared… as such" (Father b; GNHPF, RE)
Feeling foolish	Consulting colleagues	"It gives me anxiety, seeing how I take him to the emergency room, as such, if I bring him perhaps it is too early and they think: 'goodness me, this woman'… I brought him and they said: 'well, he has a huge ear infection', and I felt embarrassed" (Mother Q; GHPM, UE)
Freezing up	Not knowing how to act	"You feel uncertain of what to do" … "Then it was a feeling of impotence: 'now what do I do? What's next?'" (Mother B; GNHPM, RE)
		"I have never had 40 or 41 degrees, that is why I think that I would get overwhelmed and I wouldn't know…" (Mother S; GHPM, UE)
Suffering/ "pity"	Each child the same	"I have two sons… And you suffer just the same" (Mother J; GNHPM, UE)

		Because of the symptoms	"We had a hard time because you see him so subdued" (Mother B; GNHPM, RE)
	Obsession	Thermometers	"Obsession with the thermometer because the fever went down and half an hour later there we were again, so it became an obsession… I was so obsessed that I went to the pharmacy to find other thermometers to see whether ours was broken" (Mother K; GNHPM, UE)
	Blame	Feeling like bad parents	"It makes you feel bad because you say: darn it, as a mother I know that I am not doing things properly, poor thing, I send him there to the nursery school, where he is going to be all wiped out, but I can't miss work." (Mother T; GHPM, UE)
The need for information on fever	Source of information	Selection/screening criteria	"You have to really know where to look [for information]" (Mother E; GNHPM, RE)
		Health professional	"You take him to the pediatrician" (Father t; GNHPF, UE)
		Internet: pros/cons	"Internet is a double-edged sword" … "if you don't visit pages where information is 100% truthful, at the end every page says something and I think it can lead you to confusion" (Mother Ñ; GNHPM, UE)
		Applications for mobile phones or tablets	"The application for fever in children, it tells you, with the child's weight, it calculates the dose of paracetamol" (Mother P; GHPM, UE)
		Magazine	"... if you see a magazine" (Mother A; GNHPM, RE)
		Scientific	"I now recognize that I haven't stopped reading articles and such and that now I don't trust homeopathic medicine so much" (Mother Q;

	publications	GHPM, UE)
	Information pamphlet	"I follow the prescription" (Mother γ; GHPM, RE)
	Pharmacist	"I have asked the pharmacist many times more or less what to give him" (Father g; GNHPF, RE)
	Group of peers	"Now we have things like WhatsApp groups" … "Plenty of information from different places, your friend, so-and-so's sister-in-law… and everybody gives you their opinion" (Mother A; GNHPM, RE)
	Grandparents	"... and of course, later the grandmothers come... they overload you with information" (Mother A; GNHPM, RE)
Gaps in knowledge	Classifying level of fever	"At what point is it considered high fever?" (Mother I; GNHPM, UE)
	Fever complications	"I don't know if it affects his head" (Father d; GNHPF, RE)

(GNHPM) Group of non-health professional mothers

(GNHPF) Group of non-health professional fathers

(GHPF) Group of health professional fathers

(GHPM) Group of health professional mothers

(RE) Rural environment

(UE) Urban environment
